# Correlation between Patient-Reported Symptoms and Ankle-Brachial Index after Revascularization for Peripheral Arterial Disease

**DOI:** 10.3390/ijms160511355

**Published:** 2015-05-18

**Authors:** Hyung Gon Je, Bo Hyun Kim, Kyoung Im Cho, Jae Sik Jang, Yong Hyun Park, John Spertus

**Affiliations:** 1 Department of Cardiovascular and Thoracic Surgery, Research Institute for Convergence of Biomedical Science and Technology, Pusan National University Yangsan Hospital, Yangsan 626-770, Korea; E-Mail: jehg7332@gmail.com; 2 Department of Internal Medicine, Pusan National University Hospital and Biomedical Research Insititute, Busan 602-739, Korea; E-Mail: pons71@hanmail.net; 3 Department of Internal Medicine, Kosin University School of Medicine, 34 Amnam-Dong, Seo-Ku, Busan 602-702, Korea; 4 Department of Internal Medicine, Busan Paik Hospital, University of Inje College of Medicine, Busan 614-735, Korea; E-Mail: jsjang71@gmail.com; 5 Department of Internal Medicine, Pusan National University Yangsan Hospital, Yangsan 626-770, Korea; E-Mail: nadroj@chol.com; 6 Saint Luke’s Mid America Heart Institute and the University of Missouri-Kansas City, Kansas City, MO 64111, USA; E-Mail: spertusj@umkc.edu

**Keywords:** peripheral artery disease, revascularization, questionnaire, ankle-brachial index

## Abstract

Improvement in quality of life (QoL) is a primary treatment goal for patients with peripheral arterial disease (PAD). The current study aimed to quantify improvement in the health status of PAD patients following peripheral revascularization using the peripheral artery questionnaire (PAQ) and ankle-brachial index (ABI), and to evaluate possible correlation between the two methods. The PAQ and ABI were assessed in 149 symptomatic PAD patients before, and three months after peripheral revascularization. Mean PAQ summary scores improved significantly three months after revascularization (+49.3 ± 15 points, *p* < 0.001). PAQ scores relating to patient symptoms showed the largest improvement following revascularization. The smallest increases were seen in reported treatment satisfaction (all *p’s* < 0.001). As expected the ABI of treated limbs showed significant improvement post-revascularization (*p* < 0.001). ABI after revascularization correlated with patient-reported changes in the physical function and QoL domains of the PAQ. Twenty-two percent of PAD patients were identified as having a poor response to revascularization (increase in ABI < 0.15). Interestingly, poor responders reported improvement in symptoms on the PAQ, although this was less marked than in patients with an increase in ABI > 0.15 following revascularization. In conclusion, data from the current study suggest a significant correlation between improvement in patient-reported outcomes assessed by PAQ and ABI in symptomatic PAD patients undergoing peripheral revascularization.

## 1. Introduction

Patients with peripheral arterial disease (PAD) have greater functional impairment and faster rates of functional decline than the healthy population [1]. PAD prevalence dramatically increases with age, and most patients with PAD have one or more cardiovascular disease risk factors that should be targeted for therapy [2]. The selection of optimal medical therapy and ultimately revascularization procedure depends on quantifying PAD patients’ health status, since these interventions are primarily intended to improve symptoms, functional status, and quality of life (QoL) [3]. The ankle-brachial index (ABI) has been used as a sensitive, valuable, and cost-effective outcome measure and screening tool for PAD. However, recent publications have suggested that ABI measured by pulse palpation or automatic blood pressure devices is unreliable and correlates poorly with changes in health status scores [4]. Currently, the rates of lower extremity revascularization and related medical costs are increasing and the application of direct, patient-centered outcome assessments is necessary to quantify treatment success. The Peripheral Artery Questionnaire (PAQ) was developed to measure the health status of PAD patients based on their symptoms, functional status, and QoL [5]. We have previously translated and validated a Korean version of the PAQ in Korean PAD patients and demonstrated that PAQ scores strongly correlate with ABI [6,7]. Thus, screening programs using the PAQ in conjunction with ABI may improve the ability to detect PAD patients when assessing individuals with suspicious lower-limb symptoms. However, few studies have investigated the correlation between patient-reported symptoms and ABI following peripheral revascularization as a means to assess treatment outcomes. The aim of this study was to quantify improvement in the health status of symptomatic PAD patients undergoing peripheral revascularization via ABI and PAQ, and to investigate whether patient-reported symptom outcomes correlated with medically measured outcomes.

## 2. Results

One hundred and forty-nine PAD patients undergoing peripheral revascularization were recruited to this study. The mean age of the population was 70 ± 10 years, and 84% (*n* = 125) were men. Almost half (46%) were active or past smokers, 64% were hypertensive, 62% had dyslipidemia, and 39% were diabetic. One hundred patients underwent endovascular revascularlization. This included percutaneous transluminal angioplasty (PTA) alone of the superficial femoral and/or popliteal artery (*n* = 25), stent insertion in the iliac artery (*n* = 26)/superficial femoral artery (*n* = 6)/popliteal artery (*n* = 5), stent insertion with PTA of the superficial femoral and/or popliteal artery (*n* = 25), and PTA of the superficial femoral and/or popliteal artery (*n* = 13). Forty-nine patients underwent bypass surgery including aorto-iliac bypass (*n* = 3), ilio-femoral bypass (*n* = 3), femoro-femoral bypass (*n* = 18), and above knee femoro-popliteal bypass (*n* = 20), and below knee femoro-popliteal bypass (*n* = 5). No complications such as procedure-related death, all-cause death, major target limb amputation, and target vessel thrombosis were observed within 30 days after lower limb revascularization. Three patients developed an access-site hematoma. The baseline characteristics of enrolled patients and comparisons between percutaneous and surgical revascularization are shown in Table 1. 

**Table 1:** Baseline characteristics of enrolled PAD patients.

Variables	Total (*n* = 149)	Endovascular Revascularization (*n* = 100)	Bypass Surgery (*n* = 49)	*p* Value
Age, years	70.3 ± 9.7	70.1 ± 9.9	70.7 ± 9.3	0.704
Male, %	125 (84%)	82 (82%)	43 (88%)	0.258
Systolic BP, mmHg	125.1 ± 18.2	124.4 ± 19.8	126.7 ± 14.6	0.424
Diastolic BP, mmHg	77.8 ± 10.6	77.6 ± 10.6	78.2 ± 10.6	0.749
Heart rate, bpm	80.4 ± 13.0	80.6 ± 13.6	80.2 ± 11.9	0.870
Total cholesterol	163.8 ± 48.2	162.4 ± 53.7	166.6 ± 34.5	0.576
LDL, mg/dL	103.4 ± 47.5	105.6 ± 47.1	98.9 ± 48.9	0.568
HDL, mg/dL	40.6 ± 28.8	41.6 ± 33.9	38.5 ± 14.1	0.572
C-reactive protein, mg/L	4.2 ± 16.1	3.85 ± 15.4	0.96 ± 2.65	0.350
Ankle brachial index	0.75 ± 0.24	0.77 ± 0.24	0.70 ± 0.24	0.181
Hypertension, %	95 (64%)	63 (63%)	32 (65%)	0.465
Diabetes, %	58 (39%)	38 (38%)	20 (41%)	0.438
Dyslipidemia, %	92 (62%)	62 (62%)	30 (61%)	0.533
Current smoker, %	48 (32%)	33 (33%)	15 (31%)	0.461
Ex-smoker, %	21 (14%)	11 (11%)	10 (20%)	0.099
Aspirin, %	87 (58%)	60 (60%)	27 (55%)	0.329
Clopidogrel, %	29 (20%)	20 (20%)	9 (18%)	0.500
Cilostazol, %	10 (7%)	8 (8%)	2 (4%)	0.302
BP medication, %	88 (59%)	59 (59%)	29 (59%)	0.782
Statin, %	62 (42%)	48 (48%)	14 (29%)	0.024
Location				
Aorto-iliac	48 (32.2%)	46 (46%)	2 (4%)	<0.001
Femoropopliteal	101 (67.8%)	54 (54%)	47 (96%)	<0.001

Values are expressed as means ± SD. PAD: peripheral artery disease; BP: blood pressure; LDL: low density lipoprotein; HDL: high density lipoprotein.

### 2.1. Post-Revascularization Outcome Assessments

Post-revascularization ABI of the treated limb was significantly improved compared to baseline (Table 2). Mean PAQ summary scores also improved significantly after revascularization (*p* < 0.001). All six PAQ domains improved (Table 2). The greatest post-revascularization improvement was observed in the symptoms category, whereas the smallest increase was seen in the treatment satisfaction domain. 

**Table 2:** PAQ scores and ABI prior to and three months after revascularization.

Assessments	Pre-Revascularization	Post-Revascularization	Changes (Δ)	*p* Value
ABI in non-treated limb	0.94 ± 0.22	0.97 ± 0.16	0.02 ± 0.17	0.360
ABI in treated limb	0.65 ± 0.17	0.94 ± 0.20	0.31 ± 0.23	<0.001
Physical function	22.4 ± 12.9	81.6 ± 19.9	59.2 ± 19.8	<0.001
Symptom	22.7 ± 19.6	83.2 ± 16.7	60.5 ± 25.2	<0.001
Stability	41.6 ± 14.8	87.6 ± 19.4	46.0 ± 24.3	<0.001
Treatment satisfaction	36.8 ± 10.6	69.4 ± 16.1	32.6 ± 17.5	<0.001
Quality of life	22.1 ± 9.5	73.0 ± 18.5	50.9 ± 20.4	<0.001
Social limitation	41.8 ± 19.1	87.8 ± 15.5	46.0 ± 20.7	<0.001
Summary	32.1 ± 11.3	81.4 ± 16.4	49.3 ± 15.8	<0.001

Values are expressed as means ± SD. ABI: ankle brachial index; PAQ: peripheral artery questionnaire.

Baseline ABI of the affected limb was higher in the patients undergoing endovascular treatment compared to those having open surgery; however, post-revascularization increases in ABI were comparable between the groups. Patients receiving endovascular treatment reported a more significant increase within the social limitation domain of the PAQ than those undergoing open surgery (*p* = 0.037). No post-surgical differences were observed between the groups in the other PAQ domains, or PAQ summary score (Table 3).

### 2.2. Correlation between PAQ Score and ABI

**Figure 1:**
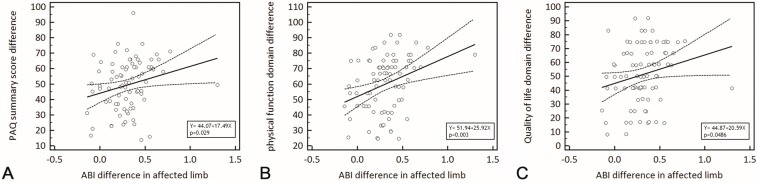
Correlation analysis assessing the association between changes in PAQ scores and change in ABI in the affected limb after revascularization. The change in summary scores (**A**); physical function (**B**); and Quality of Life domain (**C**) scores were significantly correlated with the changes in ABI.

Post-surgical scores for all PAQ domains, except for symptom stability, were significantly correlated with the post-revascularization ABI of the treated limb, as shown in Table 4. In contrast, the changes in scores from before and after revascularization were not as strongly associated with post-procedure ABIs. The change in summary score (*r* = 0.243, *p* = 0.029, Figure 1A), change in physical function (*r* = 0.326, *p* = 0.003, Figure 1B) and change in QoL (*r* = 0.220, *p* = 0.048, Figure 1C) domains showed modest correlation to the change in ABI. No significant correlation between change in symptom (*r* = 0.185, *p* = 0.098), change in symptom stability (*r* = −0.102, *p* = 0.363), change in treatment satisfaction (*r* = 0.208, *p* = 0.063) and change in social limitation (*r* = 0.046, *p* = 0.683) with change in ABI were observed.

**Table 3:** Changes in PAQ scores and ABI following lower limb revascularization via endovascular or open surgery.

Assessments	Endovascular Revascularization (*n* = 100)	Open Surgery (*n* = 49)	*p* Value
Baseline ABI in treated limb	0.68 ± 0.18	0.59 ± 0.12	0.007
Postoperative ABI in treated limb	0.96 ± 0.20	0.93 ± 0.20	0.519
ΔABI in treated limb	0.29 ± 0. 24	0.34 ± 0.19	0.391
Baseline physical function	21.3 ± 11.9	24.7 ± 14.7	0.164
Postoperative physical function	80.0 ± 21.9	84.9 ± 14.6	0.105
ΔPhysical function	58.7 ± 18.8	60.3 ± 19.2	0.641
Baseline symptom	19.8 ± 17.9	28.6 ± 21.7	0.016
Postoperative symptom	82.6 ± 17.8	84.2 ± 14.4	0.584
Δsymptom	62.9 ± 23.3	55.7 ± 28.5	0.127
Baseline stability	41.7 ± 13.9	41.3 ± 16.8	0.890
Postoperative stability	86.0 ± 20.8	90.8 ± 15.9	0.120
Δstability	44.3 ± 24.2	49.5 ± 24.4	0.224
Baseline treatment satisfaction	37.4 ± 9.7	35.6 ± 12.3	0.311
Postoperative treatment satisfaction	70.8 ± 16.0	66.7 ± 16.2	0.147
ΔTreatment satisfaction	33.3 ± 16.1	31.1 ± 20.1	0.505
Baseline quality of life	20.0 ± 7.7	26.5 ± 11.3	0.001
Postoperative quality of life	72.8 ± 16.0	73.5 ± 18.3	0.845
ΔQuality of life	52.8 ± 18.8	47.0 ± 23.0	0.125
Baseline social limitation	38.4 ± 17.2	48.6 ± 21.0	0.002
Postoperative social limitation	86.9 ± 16.5	89.6 ± 13.2	0.283
ΔSocial limitation	48.5 ± 17.8	41.0 ± 25.0	0.037
Baseline summary	30.6 ± 10.0	35.4 ± 13.1	0.025
Postoperative summary	80.6 ± 17.4	83.1 ± 14.0	0.353
Δsummary	50.0 ± 14.0	47.7 ± 19.0	0.438

Values are expressed as means ± SD. ABI: ankle brachial index; PAQ: peripheral artery questionnaire.

**Table 4:** Correlation between scores of each PAQ domain and ABI after revascularization.

PAQ Domain	Correlation Coefficient	*p* Value
Physical function	0.337	0.001
Stability	−0.122	0.231
Symptom	0.376	<0.001
Treatment satisfaction	0.427	<0.001
Quality of life	0.371	0.001
Social limitation	0.333	0.001
Summary score	0.374	<0.001

### 2.3. Differences in PAQ Scores in Patients Who Did or Did not Respond to Revascularization

**Figure 2:**
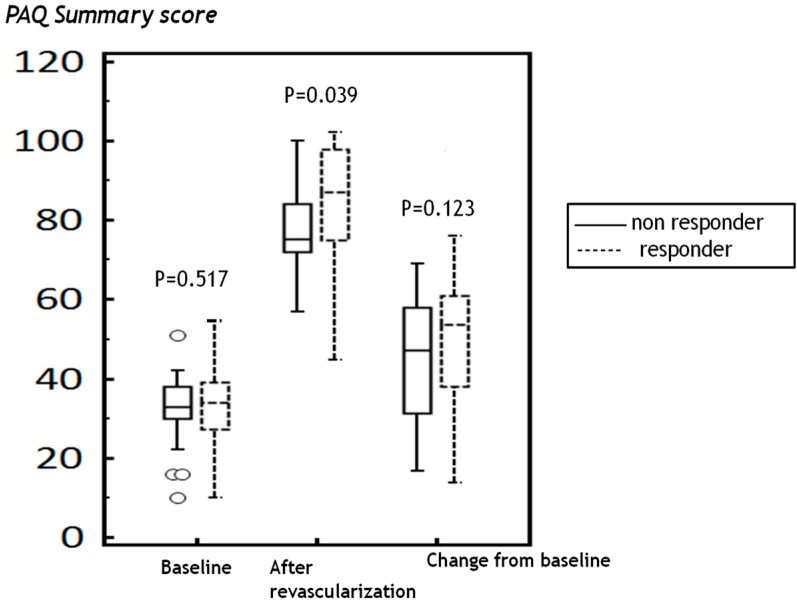
After revascularization, peripheral artery questionnaire summary scores were higher in the responder group; however, the scores from the baseline were not significantly different between the groups.

Patients were then reclassified as those who responded poorly to revascularization (post-surgical increase in ABI of the treated limb <0.15), and those who responded well (increase in ABI > 0.15). Using these criteria, 33 (22%) of patients responded poorly to revascularization. Demographic characteristics of good- and poor-responders are shown in Supplementary Table S1. Post-revascularization PAQ summary score was higher in the good-responder group (Table 5, Figure 2). Although the post-revascularization changes in PAQ scores were lower in non-responders, they were only significantly different for the treatment satisfaction domain (Table 5).

**Table 5:** Comparison of PAQ scores and ABI between responders and non-responders.

Assessments	Responders (*n* = 116)	Non-Responders (*n* = 33)	*p* Value
ΔABI, ischemic side	0.39 ± 0.18	0.02 ± 0.10	<0.001
Post-physical function	85.4 ± 16.7	77.1 ± 16.3	0.066
Post-symptom score	86.3 ± 15.2	77.3 ± 16.5	0.033
Post-stability	88.1 ± 21.5	88.9 ± 17.6	0.886
Post-treatment satisfaction	73.0 ± 14.5	63.0 ± 19.8	0.020
Post-quality of life	75.7 ± 17.9	67.1 ± 18.2	0.080
Post-social limitation	90.2 ± 14.0	81.5 ± 17.7	0.031
Post-summary score	84.4 ± 15.0	75.8 ± 16.4	0.039
ΔPhysical function	61.9 ± 18.5	53.3 ± 15.2	0.051
ΔSymptom	61.3 ± 25.3	53.7 ± 22.8	0.234
ΔStability	44.9 ± 26.0	47.9 ± 21.7	0.620
ΔTreatment satisfaction	35.5 ± 17.7	25.9 ± 18.7	0.047
ΔQuality of life	53.1 ± 20.8	44.7 ± 22.3	0.165
ΔSocial limitation	46.5 ± 20.5	43.5 ± 20.5	0.585
ΔSummary	51.0 ± 16.3	44.2 ± 16.0	0.123

Values are expressed as means ± SD. ABI: ankle brachial index; PAQ: peripheral artery questionnaire.

## 3. Discussion

In this descriptive study, we found that both ABI and PAQ showed significant improvement following revascularization therapy regardless of the surgical method. The average post-revascularization changes were 0.31 in ABI of the symptomatic limb and 49.3 points in the PAQ summary score. Comparisons between different methods of revascularization showed similar changes in ABI and PAQ summary scores, but social limitation was less compromised after endovascular revascularization. The PAQ summary score was well-correlated with ABI, and the physical function domain showed the strongest correlation. 

PAD is a chronic arterial occlusive disease of the lower extremities caused by atherosclerosis. Previous studies reported a prevalence of PAD-related symptoms (mainly intermittent claudication) ranging from 5.3% to 18.9% among the elderly population [8]. This low prevalence was attributed to elderly people not walking enough to experience symptoms, either because of impaired vascularization in the extremities or other disorders such as osteoarthritis. There is little correlation between clinical parameters of vascular disease, such as ABI, and the patient’s self-reported level of disability [9]. In fact, ABI is considered most beneficial in identifying asymptomatic individuals, rather than those with symptomatic disease being considered for intervention [3]. Another study found that most atherosclerotic events occurred in symptomatic patients with ABI < 0.9 [10,11]. 

Outcome assessments of revascularization therapy that focus on anatomic end points, such as degree of lumen narrowing and restenosis, are surrogate end points that effectively assess the physiologic impact of lower limb atherothrombosis and its treatment. These measures do not adequately describe the impact of the disease from the patients’ perspective or the benefits or adverse effects of treatment on QoL. In studies of PAD patients not receiving revascularization, ABI, a measure integrating the impact of all stenoses in a limb, is poorly associated with function [12,13]. So far, the relationship of restenosis or occlusion to symptoms and function after revascularization is poorly studied and likely to vary between individuals. Traditionally, patient-reported symptom measures and objective functional performance have been included as clinical trial end points [14,15,16,17,18,19]. Among the instruments for the assessment of patients’ symptoms, previous studies suggest that the PAQ is more sensitive to changes in patients’ health status than generic instruments, such as the EuroQol 5 Dimensions (EQ5D) [14], the health associated questionnaire (HAQ) [6], and EuroQol-Visual Analogue Scale (EQ VAS) [15]. According to our previous study, the reliability of the Korean version of PAQ was adequate with an intra-class correlation coefficient of 0.71, and Cronbach’s alpha for the summary score was 0.94, indicating good internal consistency and congruence with the original English version [6]. Recent trials have also assessed intermittent claudication-related symptoms and functional impairment using the Walking Impairment Questionnaire (WIQ) and PAQ [16]. Compared to the WIQ, which mainly focuses on the patient’s walking distance and ability, the PAQ assesses a broader range of domains, including PAD-related physical limitation, symptoms, quality of life, social function and treatment satisfaction. The clinical validity of the PAQ proved to be good in the discrimination of patients with or without symptomatic PAD and its severity as defined by walking distance [15]. Moreover, PAQ subscales were correlated with the risk factors relevant for PAD [15], and we have previously shown that the PAQ might increase the pre-test probability of ABI screening among patients with suspicious symptoms [7]. This study extends this prior work by demonstrating that the improvement in disease-specific PAQ is significantly associated with changes in ABI after revascularization.

Since the patients’ main concerns are symptom relief and improvement in daily functioning, revascularization treatment should be assessed for its success in improving patient health status [20,21,22,23,24,25]. Although ABI is known to correlate poorly with changes in health status scores [3], ABI is currently one of the most widely used tools for evaluation of peripheral revascularization. However, in patients with intermittent claudication, there is no evidence that endovascular therapy alone provides improved outcome over supervised exercise alone based on the improvement of ABI and treadmill walking [24]. Recently, six-month disease-specific QoL results in the Claudication: Exercise *versus* Endoluminal Revascularization (CLEVER) study showed that maximal treadmill walking time showed good correlation between self-reported symptom improvement in patients treated with stent revascularization, but not in patients with supervised exercise [25]. This finding indicates that traditional objective treadmill test outcomes may not correlate well with symptom relief in patients with claudication.

Considering the limited use of quality of life tools to assess outcomes in clinical practice, and the need for more patient-centered assessments of the effects of treatment, we believe the disease-specific PAQ to be the preferred choice or studying outcomes in PAD patients. However, comparison between ABI and PAQ was needed to prove clinical efficacy of PAQ-based outcome assessments after revascularization in patients with intermittent claudication. The current study firstly demonstrated that revascularization treatment resulted in significant improvements in both ABI and subjective QoL outcomes assessed by PAQ. Secondly, data show that the PAQ summary score was well-correlated with ABI in PAD patients. Changes in ABI and summary score of PAQs were similar between endovascular treatment and bypass surgery, but the improvement of social limitation was significantly higher in patients with endovascular treatment, which, if replicated, could be a benefit of endovascular treatment as compared with open surgery. 

The benefits of revascularization in patients with PAD may be closely tied to improvements in the ABI after revascularization [26]. In the current study, ~22% of patients showed a poor response to revascularization evidenced by an increase <0.15 units in ABI within the treated limb three months after surgery. Nevertheless, these “non-responders” experienced significant improvements in their health status, although not as much as the responder group. This suggests that the technical success of the operation measured by ABI failed to capture the benefits experienced by patients. Thus, the ABI change has a complementary, but not a superior, role to the PAQ in patient outcome assessment after revascularization. Conversely, a previous study showed that despite a technically successful procedure in 98% of patients, 21% of patients did not achieve the minimal clinically important improvement of an eight-point change in PAQ summary score after revascularization [14]. The reasons for the discrepancies between improvement in ABI and PAQ in the current study are unclear. 

There are several limitations of the present study. First, this is an observational study with relatively few subjects. Compared to clinical trials, however, our study comprises a rather heterogeneous population and is more representative of daily clinical practice. Second, the interval between revascularization therapy and the post-revascularization assessment of treatment was relatively short. To understand whether restenosis or disease progression affect PAQ scores will require further study. Because over 80% of participants in our study were male, the current results may not be generalizable to the correlation of ABI and PAQ in women. Finally, results of treadmill tests or other objective measures of patient symptoms like the WIQ, intermittent claudication questionnaire (ICQ), EQ-5D, PAD Quality of Life Questionnaire (PADQOL) and Vascular Quality of Life Questionnaire (VascuQoL) were not examined in the current study. No Korean versions of these questionnaires are currently available. Further large, prospective studies should be conducted to ascertain the correlation of PAQ and other objective measures of patient symptoms in Korean patients.

## 4. Experimental Section 

### 4.1. Participants and Selection Criteria

Between January 2009 and August 2012, 218 consecutive patients underwent peripheral revascularization for symptomatic PAD. To be eligible for this study, PAD participants must have been diagnosed with PAD in one of the noninvasive vascular laboratories and have an ABI ≤ 0.90 at their baseline study visit. Patients were excluded if they were below 18 years of age, refused revascularization therapy, had a history of thoracic or brain surgery, or had comorbidities such as cancer or a life-threatening illness. Among the 218 patients who underwent revascularization, 25 were excluded due to these criteria, 30 patients did not complete pre- or post-revascularization PAQ, and 14 refused to provide written informed consent. Thus, a total of 149 patients were enrolled and completed a pre- and post-revascularization PAQ, as well as post-revascularization ABI. The study protocol was approved by the Institutional Review Boards of Pusan National University Yangsan Hospital, (IRB no; 04-2011-034) and all patients provided written informed consent. Characteristics of study subjects including demographics, established cardiovascular risk factors (smoking, type 2 diabetes mellitus (DM), hypertension, or dyslipidemia, as defined by the TASC II report), family history, past medical history, history of atherothrombotic events, medications in the last six months (including anti-hypertensives, anti-diabetics, lipid-lowering drugs, and antithrombotic agents), and smoking status were obtained using a standard questionnaire. Blood pressure, heart rate, body weight, height, body mass index (BMI), waist circumference, and physical examination findings specific to PAD (absence of pedal pulses, arterial bruits, and trophic changes of the foot) were also recorded. The revascularization strategy was decided at the surgeon’s discretion. Surgical revascularization was performed using standard techniques, such as aorto-iliac bypass, ilio-femoral bypass, femoro-femoral bypass, and femoro-popliteal bypass. Femoro-popliteal bypass was performed using autologous greater saphenous vein grafts, and the extent of revascularization was left to the discretion of the surgeon. Peripheral endovascular revascularization was performed using various interventional devices, including stents. The extent of the intervention and choice of equipment were also at the discretion of the operating surgeon. Either open or endovascular revascularization was selected by consensus between the vascular surgeon and interventionist using the TASC II classification [27]. 

### 4.2. Questionnaires Administered

Patient-reported symptom outcomes were measured by the translated Korean version of the PAQ [6], a disease-specific instrument for assessing health status in patients with PAD [5,6]. The PAQ is a self-administered, 20-item health status measure for patients with PAD. Scores are separated into six domains, including physical limitation (the degree to which PAD limits the patient’s routine activities), symptoms (frequency and discomfort of intermittent claudication and the frequency of rest pain), symptom stability (recent improvement or deterioration in patient symptoms), treatment satisfaction (patient satisfaction with current treatment), social functioning (limitations in patients’ ability to interact with others), and QoL (personal evaluation that reflects the patient’s current symptoms and limitations as compared with their desired level of functioning). A summary score is calculated as the average of the physical limitation, symptoms, QoL, and social functioning scores. Scores range from 0 to 100, where higher scores indicate less functional limitation, fewer symptoms, better treatment satisfaction, higher social functioning, and better QoL. A symptom stability score of 50 represents no change over the preceding four weeks, whereas scores above or below 50 represent recent improvement or worsening of symptoms, respectively. The pre-revascularization PAQ inquired about symptoms attributable to PAD over the four weeks before revascularization, and the post-revascularization PAQ was administered three months after revascularization therapy. The changes in PAQ scores were calculated by subtracting pre-revascularization PAQ scores from the post-revascularization PAQ scores.

### 4.3. Measurement of Ankle Brachial Index

ABI was measured in all patients using an automatic waveform analyzer (VP-1000; Colin Co., Komaki, Japan) in a quiet room, with the patient supine for at least five minutes before measurement. The VP-1000 simultaneously recorded pulse waves, blood pressure (BP) in both arms and ankle, and electrocardiography. The ABI was then calculated as the ratio of the ankle systolic BP divided by the ipsilateral arm systolic BP, using the lower value of the ankle systolic BP for the calculation. The treated leg ABI was used for analysis. An increase in ABI of ≥0.15 in the revascularized leg at three months after surgery was considered a clinically meaningful ABI improvement as previously described [26]. If a participant underwent bilateral revascularization, the leg with the lowest ABI before revascularization was considered in the analysis as the lowest leg ABI most closely associated with degree of functional impairment [28]. The change in ABI was calculated as subtracting baseline ABI from the ABI after revascularization.

### 4.4. Statistical Analyses

The Kolmogorov-Smirnov test was used for the evaluation of variable distribution. Normally distributed data are presented as mean ± standard deviation, and not normally distributed data are expressed as median (inter quartile range). Categorical variables are presented as percentages or numbers. Comparisons of all measurements were made with paired *t*-tests and Wilcoxon signed-rank test for continuous variables and chi-squared and Fischer’s exact tests for categorical variables. The independent Student’s *t*-test was used to determine the difference in normally distributed data, and the Mann-Whitney U test was used for comparison of medians for not normally distributed variables. Comparison of patient-reported symptoms between responders and non-responders based on changes in ABI was analyzed with the use of independent *t*-tests. Correlations were made with the Pearson correlation test. All *p*-values were two-sided, and a probability value of *p* < 0.05 was considered a significant difference. Statistical analysis was performed using SPSS version 21.0 for Windows (SPSS Inc., Chicago, IL, USA).

## 5. Conclusions 

Our data suggest that patient-reported improvement in PAD symptoms following lower extremity revascularization is associated with, but distinct from, post-revascularization improvements in the ABI. Although patients with a post-surgical increase in ABI >0.15 showed a greater improvement in symptoms than those with ABI increases <0.15, patients who had a poor hemodynamic response to revascularization also reported important improvements in their health status. Thus revascularization can offer symptomatic relief independent of significant increases in ABI. Use of quality of life measurement tools such as the PAQ is likely to be beneficial in assessing the outcomes in PAD patients after revascularization.

## Supplementary Materials

Supplementary materials can be found at http://www.mdpi.com/1422-0067/16/05/11355/s1.

## References

[1] McDermott M.M., Liu K., Greenland P., Guralnik J.M., Criqui M.H., Chan C., Pearce W.H., Schneider J.R., Ferrucci L., Celic L. (2004). Functional decline in peripheral arterial disease: Associations with the ankle brachial index and leg symptoms. JAMA.

[2] Selvin E., Erlinger T.P. (2004). Prevalence of and risk factors for peripheral arterial disease in the United States: Results from the National Health and Nutrition Examination Survey, 1999–2000. Circulation.

[3] Mehta T., Venkata S.A., Chetter I., McCollum P. (2006). Assessing the validity and responsiveness of disease-specific quality of life instruments in intermittent claudication. Eur. J. Vasc. Endovasc. Surg..

[4] Aboyans V., Lacroix P., Doucet S., Preux P.M., Criqui M.H., Laskar M. (2008). Diagnosis of peripheral arterial disease in general practice: can the ankle-brachial index be measured either by pulse palpation or an automatic blood pressure device?. Int. J. Clin. Pract..

[5] Spertus J., Jones P., Poler S., Rocha-Singh K. (2004). The peripheral artery questionnaire: A new disease-specific health status measure for patients with peripheral arterial disease. Am. Heart J..

[6] Lee J.H., Cho K.I., Spertus J., Kim S.M. (2012). Cross-cultural adaptation and validation of the Peripheral Artery Questionnaire: Korean version for patients with peripheral vascular diseases. Vasc. Med..

[7] Kim B.H., Cho K.I., Spertus J., Park Y.H., Je H.G., Shin M.S., Lee J.H., Jang J.S. (2014). Peripheral Artery Questionnaire improves ankle brachial index screening in symptomatic patients with peripheral artery disease. Int. J. Clin. Pract..

[8] Meijer W.T., Hoes A.W., Rutgers D., Bots M.L., Hofman A., Grobbee D.E. (1998). Peripheral arterial disease in the elderly: The rotterdam study. Arterioscler. Thromb. Vasc. Biol..

[9] Feinglass J., McCarthy W.J., Slavensky R., Manheim L.M., Martin G.J. (1996). Effect of lower extremity blood pressure on physical functioning in patients with intermittent claudication. J. Vasc. Surg..

[10] Newman A.B., Shemanski L., Manolio T.A., Cushman M., Mittelmark M., Polak J.F., Powe N.R., Siscovick D. (1999). Ankle-arm index as a predictor of cardiovascular disease and mortality in the Cardiovascular Health Study. Arterioscler. Thromb. Vasc. Biol..

[11] Doobay A.V., Anand S.S. (2005). Sensitivity and specificity of the ankle-brachial index to predict future cardiovascular outcomes: A systematic review. Arterioscler. Thromb. Vasc. Biol..

[12] Atkins L.M., Gardner A.W. (2004). The relationship between lower extremity functional strength and severity of peripheral arterial disease. Angiology.

[13] Szuba A., Oka R.K., Harada R., Cooke J.P. (2006). Limb hemodynamics are not predictive of functional capacity in patients with PAD. Vasc. Med..

[14] Safley D.M., House J.A., Laster S.B., Daniel W.C., Spertus J.A., Marso S.P. (2007). Quantifying improvement in symptoms, functioning, and quality of life after peripheral endovascular revascularization. Circulation.

[15] Hoeks S.E., Smolderen K.G., Scholte O.R.W.J., Verhagen H.J., Spertus J.A., Poldermans D.J. (2009). Clinical validity of a disease-specific health status questionnaire: the peripheral artery questionnaire. Vasc. Surg..

[16] Murphy T.P., Cutlip D.E., Regensteiner J.G., Mohler E.R., Cohen D.J., Reynolds M.R., Massaro J.M., Lewis B.A., Cerezo J., Oldenburg N.C. (2012). Supervised exercise *versus* primary stenting for claudication resulting from aortoiliac peripheral artery disease: Six-month outcomes from the claudication: Exercise *versus* endoluminal revascularization (CLEVER) study. Circulation.

[17] Hiatt W.R., Hirsch A.T., Regensteiner J.G., Brass E.P. (1995). Clinical trials for claudication: Assessment of exercise performance, functional status, and clinical end points. Circulation.

[18] Murphy T.P., Soares G.M., Kim H.M., Ahn S.H., Haas R.A. (2005). Quality of life and exercise performance after aortoiliac stent placement for claudication. J. Vasc. Interv. Radiol..

[19] Bosch J.L., Hunink M.G. (2000). Comparison of the Health Utilities Index Mark 3 (HUI3) and the EuroQol EQ-5D in patients treated for intermittent claudication. Qual. Life Res..

[20] Taft C., Karlsson J., Gelin J., Jivegard L., Sandstrom R., Arfvidsson B., Dahllöf A.G., Lundholm K., Sullivan M. (2001). Treatment efficacy of intermittent claudication by invasive therapy, supervised physical exercise training compared to no treatment in unselected randomised patients II: One-year results of health-related quality of life. Eur. J. Vasc. Endovasc. Surg..

[21] Chetter I.C., Spark J.I., Scott D.J., Kester R.C. (1999). Does angioplasty improve the quality of life for claudicants?: A prospective study. Ann. Vasc. Surg..

[22] Patterson R.B., Pinto B., Marcus B., Colucci A., Braun T., Roberts M. (1997). Value of a supervised exercise program for the therapy of arterial claudication. J. Vasc. Surg..

[23] Spronk S., Bosch J.L., Veen H.F., den Hoed P.T., Hunink M.G. (2005). Intermittent claudication: functional capacity and quality of life after exercise training or percutaneous transluminal angioplasty—Systematic review. Radiology.

[24] Ahimastos A.A., Pappas E.P., Buttner P.G., Walker P.J., Kingwell B.A., Golledge J. (2011). A meta-analysis of the outcome of endovascular and noninvasive therapies in the treatment of intermittent claudication. J. Vasc. Surg..

[25] Murphy T.P., Reynolds M.R., Cohen D.J., Regensteiner J.G., Massaro J.M., Cutlip D.E., Mohler E.R., Cerezo J., Oldenburg N.C., Thum C.C. (2013). Correlation of patient-reported symptom outcomes and treadmill test outcomes after treatment for aortoiliac claudication. J. Vasc. Interv. Radiol..

[26] McDermott M.M., Kibbe M., Guralnik J.M., Pearce W.H., Tian L., Liao Y., Zhao L., Criqui M.H. (2013). Comparative effectiveness study of self-directed walking exercise, lower extremity revascularization, and functional decline in peripheral artery disease. J. Vasc. Surg..

[27] Setacci C., de Donato G., Teraa M., Moll F.L., Ricco J.B., Becker F., Robert-Ebadi H., Cao P., Eckstein H.H., de Rango P. (2011). Chapter IV: Treatment of critical limb ischaemia. Eur. J. Vasc. Endovasc. Surg..

[28] McDermott M.M., Criqui M.H., Liu K., Guralnik J.M., Greenland P., Martin G.J., Pearce W. (2000). Lower ankle brachial index calculated by averaging the dorsalis pedis and posterior tibial arterial pressures is most closely associated with leg functioning in peripheral arterial disease. J. Vasc. Surg..

