# Supplementary material for: Correlation between Patient-Reported Symptoms and Ankle-Brachial Index after Revascularization for Peripheral Arterial Disease
Source: Int J Mol Sci. 2015 May 18;16(5):11355–68. doi: 10.3390/ijms160511355 (PMC4463704; doi:10.3390/ijms160511355)
Supplement: Supplementary file 1 [file ijms-16-11355-s001.pdf]

Supplementary Information

Table S1. Demographic characteristics of the responders and non-responders.

Variables	Responders (n = 116)	Non-Responders (n = 33)	P Value
Age, years	67.1 ± 7.9	74.1 ± 9.0	0.002
Male	96 (83%)	27 (82%)	0.318
Systolic blood pressure, mmHg	127.1 ± 16.5	117.6 ± 26.9	0.169
Diastolic blood pressure, mmHg	78.0 ± 11.1	76.4 ± 5.9	0.425
Heart rate, bpm	78.4 ± 10.4	79.3 ± 14.5	0.804
Total cholesterol	171.4 ± 51.8	158.5 ± 49.6	0.362
Low density lipoprotein, mg/dL	112.6 ± 58.4	94.2 ± 42.1	0.365
High density lipoprotein, mg/dL	44.8 ± 43.4	40.9 ± 13.3	0.773
C-reactive protein, mg/L	5.32 ± 24.8	1.37 ± 2.12	0.557
Hypertension, %	73 (63%)	22 (67%)	0.365
Diabetes, %	38 (32.8%)	20 (61%)	0.012
Dyslipidemia, %	70 (60%)	22 (67%)	0.382
Current smoker, %	32 (28%)	16 (48%)	0.081
Ex-smoker, %	17 (15%)	4 (12%)	0.744
Aspirin, %	62 (53%)	25 (76%)	0.087
Clopidogrel, %	21 (18%)	8 (24%)	0.538
Cilostazol, %	8 (7%)	2 (6%)	0.677
BP medication, %	68 (59%)	20 (61%)	0.432
Statin, %	47 (41%)	15 (46%)	0.541
Location			
Aorto-iliac	38 (33%)	10 (30%)	0.379
Femoropopliteal	79 (68%)	22 (67%)	0.448
